# Do pseudo-absence selection strategies influence species distribution models and their predictions? An information-theoretic approach based on simulated data

**DOI:** 10.1186/1472-6785-9-8

**Published:** 2009-04-24

**Authors:** Mary S Wisz, Antoine Guisan

**Affiliations:** 1 Department of Arctic Environment, National Environmental Research Institute, University of Aarhus, Frederiksborgvej 399, 4000 Roskilde, Denmark; 2 Department of Ecology and Evolution, University of Lausanne, Lausanne, Switzerland

## Abstract

**Background:**

Multiple logistic regression is precluded from many practical applications in ecology that aim to predict the geographic distributions of species because it requires absence data, which are rarely available or are unreliable. In order to use multiple logistic regression, many studies have simulated "pseudo-absences" through a number of strategies, but it is unknown how the choice of strategy influences models and their geographic predictions of species. In this paper we evaluate the effect of several prevailing pseudo-absence strategies on the predictions of the geographic distribution of a virtual species whose "true" distribution and relationship to three environmental predictors was predefined. We evaluated the effect of using a) real absences b) pseudo-absences selected randomly from the background and c) two-step approaches: pseudo-absences selected from low suitability areas predicted by either Ecological Niche Factor Analysis: (ENFA) or BIOCLIM. We compared how the choice of pseudo-absence strategy affected model fit, predictive power, and information-theoretic model selection results.

**Results:**

Models built with true absences had the best predictive power, best discriminatory power, and the "true" model (the one that contained the correct predictors) was supported by the data according to AIC, as expected. Models based on random pseudo-absences had among the lowest fit, but yielded the second highest AUC value (0.97), and the "true" model was also supported by the data. Models based on two-step approaches had intermediate fit, the lowest predictive power, and the "true" model was not supported by the data.

**Conclusion:**

If ecologists wish to build parsimonious GLM models that will allow them to make robust predictions, a reasonable approach is to use a large number of randomly selected pseudo-absences, and perform model selection based on an information theoretic approach. However, the resulting models can be expected to have limited fit.

## Background

Species distribution models (SDM) [1] are increasingly used in many fields of ecology and evolution. They have been used to address fundamental questions such as those exploring macroecological patterns [2,3] and to address applied issues such as ecological impacts of climate change or biological invasions. These tools relate field observations to environmental predictor variables, based on statistically or theoretically derived response surfaces, for prediction and inference [1]. Two groups of techniques are generally used. Techniques that require data documenting the species presence only are called "profile techniques" while those that require both presence and absence data are called "group discrimination techniques" [4]. Examples of profile techniques include BIOCLIM [5], DOMAIN [6], Species-PCA [4], and Ecological Niche Factor Analysis: ENFA [7]. Their development and use has been stimulated by the many presence-only data available in existing natural history collections [8]. Group discrimination techniques are derived from established statistical approaches and are more numerous than profile techniques. They include classical regression-based approaches such as generalised linear and additive models [9,10] but also more recent and robust techniques such as boosted regression trees (e.g. BRT; [11] or random forest:RF [12]). See [13] and [1] for a more exhaustive overview of existing approaches.

Among group-discrimination techniques, logistic regression modelling (LRM), a particular branch of generalized linear models (GLM) for binary responses, remains the most widely used so far to predict the potential distributions of species [10]. Based on a well established body of statistical theory, it is possible to do all of the following within the LRM framework: 1) construct a parsimonious model that strikes a balance between bias and variance using criteria supported by an established body of statistical theory, 2) identify the relative importance of the predictor variables, 3) explore and interpret the response of the species to each predictor, 4) estimate the uncertainty associated with parameter estimates, 5) predict the probability of observing the species (rather than predicting binary presence-absence) and 6) explore spatially explicit patterns of uncertainty in predictions.

### Fitting models with pseudo-absence data

Despite its numerous advantages, LRM has been precluded from many studies of species distributions because it requires absence data, which are frequently unavailable and often not reliable. This is an acute problem for the study of poorly documented, cryptic, rare or highly mobile species [8], many of which may be of special conservation interest. In order to facilitate the use of LRM when absence data were unavailable, a number of studies have used pseudo-absences in place of real absences [14-21].

### Techniques for generating pseudo-absences

#### Random pseudo-absences in group discrimination techniques

A basic technique for generating pseudo-absences selects them at random from the study area. A large international experiment with robust independent evaluation data [13] showed that using group-discrimination techniques with random pseudo-absences out-performs predictions made by profile techniques (including BIOCLIM and DOMAIN). Hence, given an adequate set of presence locations, models generated with random pseudo-absences can yield useful results. A potential drawback to using random pseudo-absences is that pseudo-absences might coincide with locations where the species actually occurs. This affects the calculation of probability of presence, and consequently, models built with random pseudo-absences are expected to have poorer fit, and lower predictive performance than models built with real absences.

#### Pseudo-absences from two-step modelling

In an attempt to overcome these drawbacks, two-step modeling approaches have been implemented to restrict pseudo-absences to locations expected to have a low habitat suitability according to a preliminary model. For example Engler *et al. *[18] used a two step modelling approach to predict the distribution of a rare plant in Switzerland. In the first step, they used the profile technique "ENFA" to map potential habitat suitability for the species, and then selected pseudo-absences from the areas predicted to have low suitabilty. They subsequently included the pseudo-absences in a second logistic regression model which was used to predict the final species' potential distribution and prioritize further field work. The choice of profile method is a potentially critical step as this can result in very different predictions, and may have implications for results. Among other reasons, predictions can vary depending on the way profile methods (such as BIOCLIM, DOMAIN, ENFA or S-PCA) handle predictor variables [22]. Some weigh them equally, such as BIOCLIM or DOMAIN, while others, such as ENFA and S-PCA weigh the variables according to their fit. Thus two-step pseudo-absence selection may add noise to the modeling process by introducing additional uncertainty and bias. We expect that the choice and implementation of any profile model used to stratify the selection of pseudo-absences will influence parameter estimates, and weaken fit and predictions of the subsequent group-discrimination model.

### Testing with a virtual species

It is unclear which pseuduoabsence selection method should be the most appropriate for modeling species' distributions. It is also difficult to address this question with real data due to the complexity of the modeling process and the potential for introducing noise at each stage. Consequently, a growing number of studies have used virtual species in real or artificial landscapes to study the behavior of different implementations of predictive distribution models [23-27]. In a virtual species approach, the species' distribution is defined *a priori*, by specifying its ecological niche as a simple mathematical relationship to the set of predictor environmental variables (e.g. in the form of a multiple logistic regression equation) and projects this relationship onto a map of the study area to define its "true" distribution. One can then attempt to recover the virtual species' known distribution by building models from samples drawn from the study area and then evaluate the efficacy of different implementations of models by comparing their parameter estimates and predictions to the true distribution of the species. Such an approach has never been used to assess the best way to select pseudo-absences.

### Aims of the study

Using a simulated, virtual species in which a true distribution is known in a real landscape, we used a robust model selection framework proposed by Burnham and Anderson [28] to addresses two main questions: (1) How does the selection of pseudo-absences influence variable selection, model fit and predictions of a single simulated species? (2) Which pseudo-absence strategy yields a prediction most similar to the predefined real distribution (i.e. "truth")? We expected the following: True absences will yield models with the best fit, predictive power, and the correct model (the one including the correct predictors and parameter estimates) will be the best-supported by the data, as indicated by Akaike's information criterion (AIC). Pseudo-absences selected from a profile model within a two-step modeling approach (as described below) should yield the next-best models in terms of model fit and predictive power. However, due to the noise introduced by the preliminary profile model the correct model may not be the best-supported by the data. Random pseudo-absences should yield the worst fitting models with the lowest predictive power, and models that include uninformative predictors may even be supported by these data.

## Results

### LRM models with pseudo-absences and the 3 "correct" predictors

#### Fit of selected models

**Figure 1:**
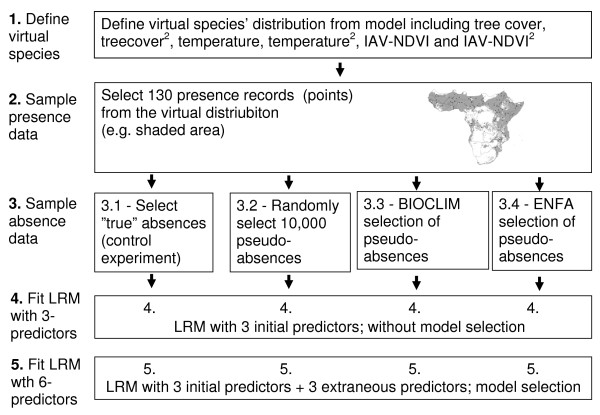
**Chart summarizing methods**.

**Figure 2:**
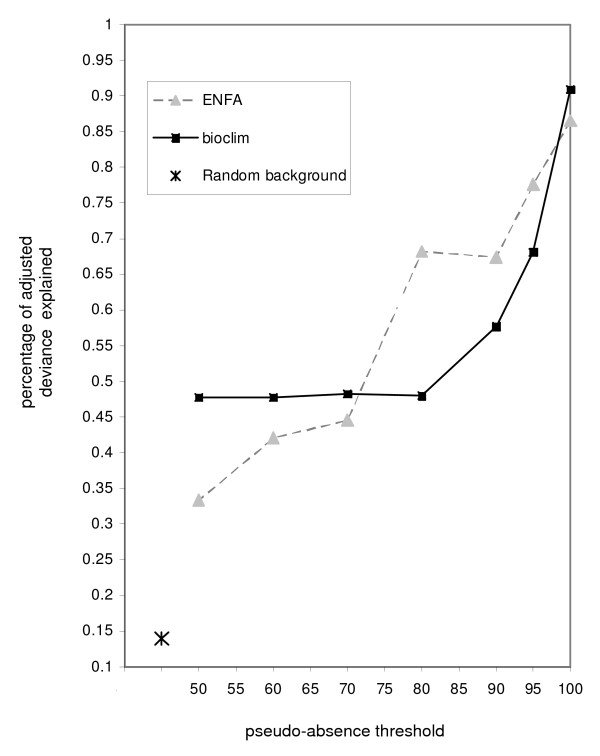
**Percent adjusted deviance explained by models developed using 2 step and random pseudo-absence strategies (see bottom left corner of plot)**. Each model included the same three predictors used to define the virtual species distribution (tree cover, IAVNDVI, and minimum average temperature) along with their quadratic expressions.

**Figure 3:**
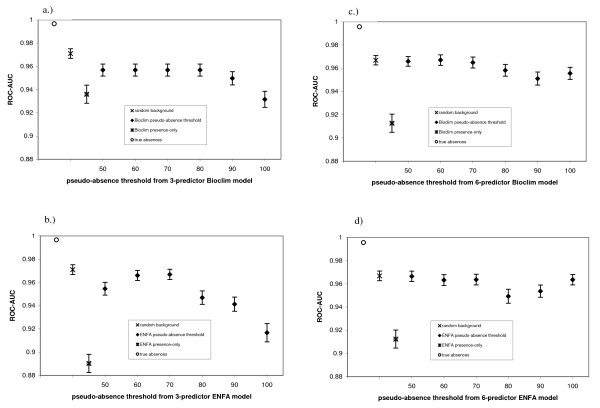
**ROC-AUC values assessing model discriminatory power for each pseudo-absence threshold from 3- predictor models (correct predictors) including (tree cover, IAVNDVI, and minimum average temperature) along with their quadratic expressions (a-b), plus 6 predictor models that included these plus 3- incorrect predictors including minimum NDVI, seasonality of precipitation, and elevational range (c-d)**. Model selection was performed using model averaging based on AIC (c-d).

Adjusted deviance using the 3 correct predictors (based on methodology summarised in Figure 1) was highest for the model that used correct (i.e. true) absences (0.987) (Figure 2). For both two-step pseudo-absence methods (BIOCLIM and ENFA), the adjusted deviance declined from over 80% for the most inclusive pseudo-absence thresholds (e.g. 100^th ^percentile) to below 50% for the least inclusive thresholds (e.g. 50^th ^percentile) (Figure 2). The random background pseudo-absence selection strategy yielded the lowest proportion of adjusted deviance explained (14%) (Figure 3).

#### Model predictive power

**Figure 4:**
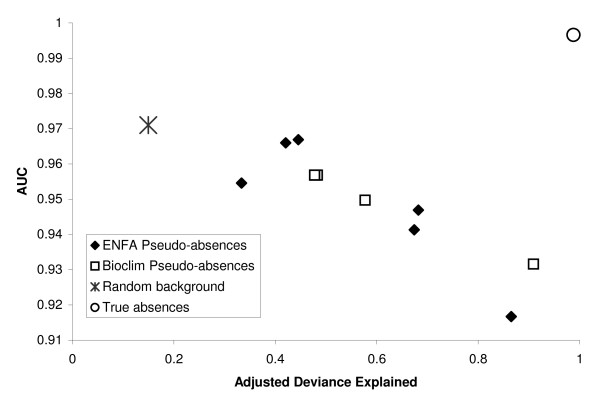
**ROC-AUC (discriminatory power) for models built only with the 3 correct predictors versus adjusted deviance explained (i.e. model fit)**. Model fit and discriminatory power are not always inversely correlated. The model built with "true" absences achieved high values for both. Thus ROC-AUC and adjusted deviance measure very different aspects of a model's performance and one should never be used as a surrogate for the other.

AUCs for predictions resulting from one-step profile methods (BIOCLIM and ENFA), were lower than those obtained from LRMs. The LRM that used correct (i.e. true) absences achieved the highest AUC, and lay well above the 95% confidence intervals of the other methods. AUC for the completely random pseudo-absence strategy was clearly better than using pseudo-absences from any two-step approach from BIOCLIM and also better than most two-step approaches using ENFA. Among the two-step approaches, the least inclusive thresholds (e.g. 100^th ^percentile thresholds for ENFA and BIOCLIM) exhibited the lowest AUC values. The GLMs that included the incorrect predictors yielded AUC values higher than those that included only the 3 correct predictors. (Figure 4)

#### Relationship between explanatory and predictive power

AUC values and adjusted deviance values were not positively correlated (Figure 4). Typically, models that had high adjusted deviance had lower AUC values based on independent data. One particular exception is the model that used correct (i.e."true") absences. Both its adjusted deviance and AUC were near one. (Figure 4)

### LRMs including 3 correct predictors and "noise" predictors: ranking models based on AIC

The correct model (i.e. the one that included only the 3 true variables) did not have the lowest (i.e. best) AIC value, even when true absences were used (Table 1). When true absences were used, the true model was ranked second after the best model, and also received considerable support by the data (delta AIC = 1.31). Nevertheless, the Akaike weights were very small for all the candidate models, indicating high model selection uncertainty. The true model was ranked seventh in the experiment that used random pseudo-absences, and this model also received considerable support by the data (delta AIC = 1.47). Whenever a two-step modelling approach was used, the true model was only weakly supported by the data, as AIC values were never 2.0 or less.

**Table 1:** AIC-based calculations for the various pseudo-absence strategies.

Pseudo-absence Selection strategy	Ranking of true model out of 726 candidate models based on Akaike weights (w_*i*_)	Delta AIC (Δ*AIC*) of the true model	Akaike weight (*w*_*i*_) of the true model	Akaike weight (w_*i*_) of the top ranking model
ENFA				

100	9	3.604723	0.023729	0.143891
90	42	6.920832	0.004007	0.127529
80	16	3.882684	0.018746	0.130621
70	14	3.845144	0.021403	0.146365
60	13	3.863541	0.021315	0.14711
50	12	3.737772	0.025063843	0.162440578

Bioclim				

100	55	9.80294298	0.001365089	0.183588
90	22	3.599437	0.013378	0.080906
80	33	3.027241	0.009334	0.042406
70	38	3.581413	0.007646	0.045825144
60	26	3.146754	0.0108	0.052088
50	33	3.708884	0.008264	0.052791
Random	7	1.474885235	0.034985305	0.073139658
True absences	2	1.130481	0.104319	0.183588

For each pseudo-absence strategy we fit 726 candidate logistic regression models using 131 presence records and 10,000 pseudo-absences. (ΔAIC) values less than 2 indicate considerable support, > 2 but < 10 indicate substantially less, and > 10 indicate essentially no support by the data. Akaike weights (*w*_*i*_) can be interpreted as the percent of times we would expect a candidate model to be the one most strongly supported by the data if the experiment were repeated on a different sample.

## Discussion

### On the use of simulated data

The primary goal of this paper was to determine how the choice of pseudo-absence strategy influences variable selection, model fit, and the discriminatory power of predictions on independent sites in a virtual species where the "true" (i.e. correct) distribution is known. Evaluating so many aspects of model's behaviour and performance is facilitated through use of a virtual species, because a real species' "true" relationship to environmental variables is never known. In the real world, sufficient amounts of reliable, completely independent, presence and absence data are rarely available to evaluate the predictive power of complex models in a controlled manner. Our virtual species' distribution was defined exclusively by a limited number of climatic factors and was not complicated by spatial auto-correlation, biotic interactions or history. This ensured that variability in model performance resulted from pseudo-absence selection, and not by omission of important predictor variables into the model (i.e. missing explanatory variables for capturing autocorrelation, biotic, or historical influences).

### Profile methods versus group discrimination techniques

Although, profile models using BIOCLIM and ENFA still performed reasonably well in terms of AUC, they were outperformed by the multiple logistic regression models using random pseudo-absences. This is in line with recent results showing that profile techniques (BIOCLIM, DOMAIN and LIVES) did not perform as well as group discrimination techniques (including GLM and several other regression-based methods) when evaluated on robust independent data [13].

BIOCLIM models that included 3 predictors performed better than those that included 6 predictors, possibly because the noisy variables constrained the bioclimatic envelope too much, resulting in an underprediction of the species' distribution. ENFA models built with 3-predictor showed somewhat lower discriminatory power than 6-predictor ENFA models, which is likely to result from adding noisy variables that are correlated with the correct (i.e. true) variables and explain some additional variance through spurious effects, a situation likely to occur also with real species.

A large body of statistical literature suggests that parsimonious models, including a small number of predictor variables, should have greater predictive power to independent samples than models including more predictors ([29-32]. Neither ENFA nor BIOCLIM are based on this principle (i.e. they do not incorporate way to select predictors), and both showed somewhat lower predictive power in terms of AUC than group discrimination models based on pseudo-absences. Real species' response shapes to explanatory variables are likely to be better represented by unimodal or even skewed functions than by rectilinear envelopes [33], and our virtual species was defined to have non-linear relationships to predictors. BIOCLIM and ENFA cannot incorporate quadratic relationships to predictors, so this may be another reason the GLM methods performed better.

The response curves from GLM, including simple polynomial expansions as we have included here, also offers a more flexible representation of species' ecological niches, and thus of their associated geographic distributions. Provided that data are sufficient to adequately describe these relationships correctly, they should, in theory, yield more realistic models. Indeed, with small datasets, models for which complexity is calibrated for sample size, such as MAXENT ([34] have better predictive power than models that use complex response shapes regardless of sample size (Wisz et al. 2008).

All of our predictions achieved high AUC scores. This was expected due to the fact that the three variables used to generate the virtual species' distribution were present in all the models, and deliberately entered in the same order as the "true" model. Thus, our results confirm the importance of careful selection of predictor variables [28]. Another factor that may have contributed to the high predictive power is that our records were randomly scattered throughout the range of the species, which may have provided adequate sampling across the range of habitats where it occurs. Random stratified samples may have improved this further, as they typically result in models that predict with higher accuracy [25] however these are infrequently used in practice [35,36].

### Influence of pseudo-absence selection

Through the use of a simulated species we confirmed that although randomly selected pseudo-absences yield models with lower fi to the training data, they outperform models developed from two-step methods in terms of predictive power and variable selection. Thus randomly selected pseudo-absences may be a reasonable alternative when real absences are unavailable.

Consistent with our predictions, our findings confirm that true absences outperform any pseudo-absence selection strategy. The most parsimonious model based on true presences and absences had the best fit to the data and the highest AUC scores. Because of the simplicity of our virtual species example, we were able to show that a two-step pseudo-absence selection approach does not improve a models' discriminatory power over random pseudo-absence selection, and that both can provide rather high model evaluation results. This contrasts with the findings by [18], who reported lower AUC scores and maximum kappa for randomly selected pseudo-absences than for pseudo-absences selected using ENFA. However, because their approach was applied to a rare species, using a very limited number of available occurrences and no knowledge of the true distribution, they needed to assess the discriminatory power on the same data set used to develop the model. As they pointed out, this is a less rigorous test of the model's predictive performance than using a completely independent dataset of real presences and absences, as done here. The better fit of their two-step model is therefore not surprising, as corroborated by our results. We also found that two-step pseudo-absences result in models that fit the data better than random pseudo-absences, however we also found that two step pseudo-absences result in models with weaker predictive power because they lead to overfitting. An overfit model will always have a higher value of adjusted deviance than a simpler model nested within it, but its predictive power to an independent sample will be lower because the model losses generality.

Nevertheless, our results confirm a different finding of Engler *et al. *[18]: pseudo-absence selection clearly influences model fit as measured by the percent of deviance explained. We expect this result to be continuously supported in future work, because randomly selected pseudo-absences cannot be prevented to fall in locations that are ecologically extremely similar to presence locations, which makes it more difficult for regression algorithms to estimate the model parameters.

#### Variable selection based on an information theoretic approach

In our model selection experiment, the true model received considerable support by the data in a comparison of delta AIC values, but was not selected as the AIC best model when ranked against all other candidate models. We believe this is because although the "true" model in our list of candidate models consisted of the correct predictor variables, its parameters had to be estimated using maximum likelihood methods from 130 presence records and 10,000 absences balanced using case weights, both of which probably affected the model coefficients By sampling only a limited amount of presences we introduced bias, which probably explains why the true model was not fully recovered. However, using real absences, the "true" model was ranked second and had a delta AIC value of 1.13, indicating considerable support by the data. The top ranked model only differed by missing the quadratic term for temperature, which had a very low value in the initial model (0.0001). It is likely that the sampling of a limited number of species occurrences (*n *= 130) contributed to decrease the fit of this predictor.

## Conclusion

Approaches that combine logistic regression in an information theoretic framework [30] facilitate the exploration of relationships between the probability of species' occurrence and individual predictor variables in parsimonious models that account for uncertainty in parameter estimates and model selection. This offers clear advantages over methods that do not incorporate parsimony or allow for such rigorous quantification of uncertainty. Logistic regression methods have not been widely used in many ecological contexts because they require absence data which are usually not available. Our results confirm that robust models can be generated with logistic regression using randomly selected pseudo-absences, and that such models outperform profile techniques and two-step modelling approaches that use intermediate models to select pseudo-absences. Moreover, using a virtual species in a real landscape, we confirm that simulated data represent a powerful approach to provide unambiguous answers to methodological questions. In cases where sufficient real absence data are not available in real ecological datasets, our results encourage further use of randomly generated pseudo-absences with logistic regression analysed within an information theoretic framework.

## Methods

### Methodological overview

First, we simulated a virtual species whose geographic distribution and relationship to 3 explanatory variables was defined *a priori*. Next, we built and compared multiple logistic regression models (LRM; i.e. GLM with binary response) fitted using: 1) true absences (as a control experiment), 2) completely random pseudo-absences, and 3) pseudo-absences selected from two-step approaches where unsuitable habitat was defined *a priori *from a profile method (BIOCLIM or ENFA). Finally, we repeated this procedure using extraneous (i.e. noise) predictor variables along with the true predictors, and used an AIC-based model-selection approach to evaluate how different pseudo-absence selection strategies affected the selection of candidate models (Figure 1)

### Analytical steps

There were 5 main steps in our analytical approach. Specific statistical details are provided in the following section.

#### Step 1 – Generating the virtual species' niche and distribution

We generated a simple ecological niche for our virtual species in a real landscape using a seven-term model consisting of an intercept plus three linear environmental variables (*x*) and their quadratic expressions (*x*^2^). The real landscape used in this study is the same portion of Sub-Saharan Africa as applied in continental scale macroecological work [3]

We used the formula for a binary logistic regression model

(1)

where *P *is the probability of observing the virtual species, and

(2)

where treecover is the percent tree cover estimated by the MODIS satellite [37] across sub-Saharan Africa, productivity is the inter-annual variability of NDVI estimated across 17 years, and temperature is the coldest month mean temperature. Throughout the rest of this paper, we will refer to this set of 3 predictors as "correct" variables. We then projected this formula, which defines the species' niche, onto geographic space to obtain, after inverse-logit transformation, the true virtual species' distribution map on a probability scale (0 to 1). This map of continuous probability values was then converted into a binary map, by applying an arbitrary threshold of 0.5, to mimic a real presence/absence pattern.

#### Step 2 – Selecting presences to fit the models

From the virtual species distribution generated in Step 1, we randomly selected 130 presence records, as this number borders on typical for some moderately well-known species in many of the databases we have worked with. It is also in keeping with the recommended benchmark sample size for regression analyses containing 13 terms in a model. Typically, the number of predictor terms should not exceed *n*/10 in regression analysis, where n is the least represented category in logistic regression with a binary response variable [28,38].

#### Step 3 – Selecting absences and pseudo-absences

Four different types of absences were sampled to fit the models as follows:

3.1 – As an experimental control, we selected 10,000 "true" absences from outside the species' known range.

3.2 – We selected 10,000 pseudo-absences randomly from the map of the study area. Here, pseudo-absences were allowed to fall within the true distribution of the species, but did not overlap with the 130 presence sample points

3.3 – We fitted a BIOCLIM model using the 130 presence records selected in Step 2 and the same three linear predictors used to generate the virtual species. The resulting prediction map was then used to stratify pseudo-absence selection from the least suitable locations. We converted these continuous predictions to binary presence-absence maps by applying a threshold before drawing the pseudo-absences. As this step can have a strong influence on further results, multiple thresholds were tested. For BIOCLIM, they corresponded to the percentiles: 100^th^, 90^th^, 80^th^, 70^th^, 60^th ^and 50^th^. For example, to select pseudo-absences for the 90^th ^percentile, all of the pseudo-absence were selected from outside the 90% bioclimatic envelope (i.e. the tightest-fitting envelope in environmental space that contained 90% of the observed presences).

3.4 – As 3.3, but using ENFA instead of BIOCLIM as predictive technique to select pseudo-absences from areas predicted to have the lowest habitat suitability score for the species. With ENFA, six habitat suitability thresholds were identified that included different percentages of the 130 presence locality records: 100%, 90%, 80%, 70%, 60% and 50%. In this case, the 90% threshold was that which included 90% of the locality records that had the highest ranking predicted suitability.

#### Step 4 – Fitting 3-predictor logistic regression models (LRM)

4.1 – Using the 130 true presences and the 10,000 true absences (weighted to ensure equal prevalence with presences), we fitted a LRM including the 3 "correct" predictor variables (see Equation 2). No model selection was involved in this step. Results of this step show how a random sample of true presences and true absences affects model fitting and predictions.

4.2 – Same as 4.1, but using random pseudo-absences obtained in step 3.2.

4.3 – Same as 4.1, but using BIOCLIM pseudo-absences obtained in step 3.3.

4.4 – Same as 4.1, but using ENFA pseudo-absences obtained in step 3.4.

#### Step 5 – Fitting 6-predictor logistic regression models (LRM) with selection

5.1 – LRMs were fitted by relating true presences and true absences to the three original predictors plus three extraneous (noise) predictors: seasonality, elevation range, and productivity during the driest month. Correlations between these predictors are presented in Table 2. We entered the terms for the three true predictors first into the models, in the same order as they were used to define the virtual species' distribution, subsequently adding the three noise predictors in the formula.

**Table 2:** Spearman rank correlation coefficients (*r*) of the predictors.

	**IAV-NDVI**	**Minimum Temperature**	Seasonality of Precipitation	Mimimum NDVI	Elevational Range
**Percent Treecover**	0.12	0.32	-0.62	0.79	0.03
**IAV-NDVI**		0.08	-0.2	-0.05	0.19
**Minimum Temperature**			-0.10	0.12	-0.01
Seasonality of Precipitation				-0.66	-0.24
Mimimum NDVI					0.16

The first three predictors (percent tree cover, IAV-NDVI, and minimum temperature, in bold) were used to define the distribution of the virtual species.

5.2 – Same as 5.1, but using random pseudo-absences obtained in step 3.2.

5.3 – Same as 5.1, but using BIOCLIM pseudo-absences obtained in step 3.3.

5.4 – Same as 5.1, but using ENFA pseudo-absences obtained in step 3.4.

### Statistical details

#### BIOCLIM modelling

In BIOCLIM, all explanatory variables are assigned an equal weight in the analysis, and the value of each environmental variable at the location of a species' occurrence record is used to calculate a percentile distribution for each environmental variable. For example, the 90th percentile contains the central 90% of the observations for all variables. The 10% of values outside that limit (i.e. the tails of the distribution) are mapped outside the 90% environmental envelope [5]. BIOCLIM output typically consists of four groups: outside the observed distribution, and the 100, 95 and 90 percentiles. To increase the sensitivity of our analysis, we mapped these as well as the 80, 70, 60, and 50 percentile scores using an Avenue script for ArcView 3.2.

#### ENFA modelling

*Ecological niche factor analysis *(ENFA; [7] is a profile technique that apportions a weight to each explanatory variable according to its explanatory power, and computes a habitat suitability value for each grid cell in the study area that is proportional to the distance between their position and that of the species centroid in a multidimensional environmental space. We performed all ENFA modelling using Biomapper vers. 2.1 software [39] after normalizing the environmental layers through a recommended Box-Cox transformation [7]

#### Logistic regression modelling (LRM) and model selection

Model selection, the process of selecting predictors and parameter estimates in a model, is considered one of the most crucial steps in a model building procedure [40], and model selection in logistic regression can be performed in a variety of ways. Stepwise variable selection procedures are widely used [41], but they are known to be very sensitive to small perturbations of the response data, which can lead to the selection of vastly different variables as the data are subsetted [40]. This introduces noise through model selection uncertainty [29]. An alternative to stepwise procedures is a widely used tool rooted in the principal of parsimony and uses Akaike Information Criteria (AIC; [42]) to rank the quality of models by a quantitative measure of model fit and number of parameters. Within a set of candidate models, those with relatively low AIC values are the most parsimonious and strike a better balance between bias and variance of model predictions. AIC is a measure of the information lost in using a particular candidate model to approximate a model that is theoretically true.

#### Information-theoretic approach

Information-theoretic methods of model selection rely on the calculation of the Akaike Information Criteria (AIC) [42] as a model selection tool. Within a set of candidate models, models with relatively low AIC values are the most parsimonious and strike a balance between bias and variance of model predictions. AIC is a measure of the relative Kullback-Leibler information lost in using candidate model *i *to approximate truth *j*.

(3)

where log_*e*_(*ℓ*(*θ*|*data*)) is the value of the maximized log-likelihood over the estimated parameters given the data and the model, and *K *is the number of parameters in candidate model *i*.

When n/K is less than 40, where *n *is sample size and *K *is number of parameters, Burnham and Anderson [28] recommend using a small sample size corrected version of AIC called AICc.

(4)

For each of the candidate models, we calculate AIC (or AICc) and then rescale these values to calculate AIC differences (Δ_*i*_) so that the model having the lowest AIC (or AICc) value has a Δ_*i *_value of 0, i.e.

(5)

where AIC*i *is the AIC (or AICc) value of the *i*th model, and minAIC is the AIC (or AICc) value of the model with the lowest AIC (or AICc) value. Thus, the model with a (Δ_*i*_) = 0 is the Kullback-Leibler best approximating model in the candidate set. The larger the value of (Δ_*i*_), the less plausible is the fitted model *i *as being the best approximating candidate model. Typically, models with (Δ_*i*_) values between zero and two have strong support. Models with (Δ_*i*_) values between two and ten have considerable support, and models with (Δ_*i*_) values larger than ten have essentially no support [28].

From the Akaike differences (Δ_*i*_), one can derive the Akaike weights (*w*_*i*_) for each of the *r *candidate models.

(6)

Akaike weights (*w*_*i*_) approximate the probability that a given candidate model will be the Kullback-Leibler best model (best approximating model in the set of candidate models) if the analysis was repeated on a different sample drawn from the population. These weights are scaled between zero and one, and represent the evidence for a particular model as a proportion of the total evidence supporting all of the models. Therefore, all Akaike weights sum to one, and a model with a Akaike weight of 0.9 is expected to be the Kullback-Leibler best model in 90% of all possible samples. The candidate model with the largest Akaike weight is the most parsimonious model and has the most support among the specified candidate models, given the data.

However, more than one model may be supported by the data. In such a case it is possible to calculate a composite model that is a weighted average of all the candidate models. In such an instance we can compute new parameter estimates for each term in the global model by weighting them by the Akaike weights

(7)

Where  is the model averaged parameter estimate based on all *R *models, (*w*_*i*_) is the Akaike weight for a given candidate model *i*, and  is the parameter estimate for a term in a given candidate model. The parameter estimate for terms that do not feature in a candidate model but are present in the global model is taken to be zero.

#### Model selection including correct and incorrect variables

Truth is unknown except in simulation studies, such as ours employing a virtual species, but a property underpinning of the theory of AIC is that it should select the model in the set of candidates that is closest to "truth" [42]. As an alternative to stepwise procedures, which would have introduced unmanageable noise into our analyses by introducing model selection uncertainty, we computed AIC for each of 726 multiple logistic regression candidate models. These models represented all possible combinations of the 3 linear predictors and their quadratic forms (determinants) used to define the virtual species distribution (percent tree cover, inter annual variability of NDVI, and minimum temperature), plus the 3 incorrect predictors (i.e. those not use to define the virtual species' distribution) and their quadratic expressions. Each candidate model included an intercept. For each candidate model, we computed Akaike's Information Criterion for small sample size (AICc), Delta AIC (ΔAIC) and Akaike Weights (*w*_*i*_), and an average model where the parameter estimates were calculated as an average of all candidate models weighted by each *w*_*i*_, which was then used for spatial prediction. Calculations were made in S-plus using a set of custom model averaging functions that we developed based on theory presented in Burnham and Anderson [28].

#### Comparing model fit and predictive performance

We estimated model fit of the final LRM models using the percent adjusted deviance explained, which is a sample size-adjusted measure the goodness of fit of a GLM. We also evaluated how well the model could distinguish between presence and absences (model discriminatory power) using calculations of the Area Under the Curve (AUC) of a Receiver Operating Characteristic Plot [43]. We calculated AUC for all BIOCLIM, ENFA and all LRM models by comparing their predictions to 5000 randomly selected presence/absence locations throughout the virtual species range. These 5000 records were not used to build any of the models, but represent a set of independent locations for testing the models' predictions. AUC is an appropriate metric for evaluating classification accuracy in species distribution models because it estimates the percentage of locations where the species is observed to be present that are expected to have a higher predicted probability of occurrence than places where the species is absent [43]. An AUC score of 0.8 indicates that in 80% of all locations being evaluated the model predicts higher where the species is present than where it is absent. Moreover, AUC is a threshold independent metric, which means it assesses classification accuracy across the entire range of predicted probabilities, and not just for a specified probability threshold. Recent work has called attention to problems associated with using AUC to compare models derived for multiple species though it remains a valid measure for comparing models developed for the same species in a fixed study area [44], such as ours. We calculated AUC and its 95% confidence intervals using an S-plus script modified from existing codes [45].

## Abbreviations

AIC: Akaike's Information Criterion; AUC: Area Under the Curve (Receiver operating characteristic curve); ENFA: Ecological Niche Factor Analysis; GLM: Generalised Additive Model; IAV-NDVI: Inter-Annual Variability in NDVI (a vegetation index); LRM: Logistic Regression Model.

## Authors' contributions

MSW implemented the statistical analyses for this paper. MSW and AG contributed equally to the idea, design of the study, interpretation of the results, and writing. Both authors read and approved the final manuscript.
